# Effect of staining techniques and repeated firing cycles on translucency, color and biaxial flexural strength of advanced lithium disilicate containing Virgilite crystals

**DOI:** 10.1186/s12903-025-06011-4

**Published:** 2025-05-05

**Authors:** Nadine I. Rizkallah, Ghada Abdelfatah, Marwa M. Wahsh, Hoda M. Abdel Sadek

**Affiliations:** 1 Department of Fixed Prosthodontics, Faculty of Dentistry, Ain Shams University, Cairo, Egypt; 2 Department of Fixed Prosthodontics, Faculty of Dentistry, Ain Shams and Galala University, Cairo, Egypt; 3 Faculty of Dentistry, Ain Shams University, Organization of African Unity St, El-Qobba Bridge, El Weili, Cairo, Egypt

**Keywords:** Advanced lithium disilicate, Translucency, Flexural strength, Repeated firing, Staining technique

## Abstract

**Background:**

The repeated firings can enhance shade matching, translucency, and strength; however, they may also lead to color shifts. Previous research suggests that multiple firings enhance these properties to a certain extent; however, the impact of staining techniques remains underexplored. The aim of this study is to investigate the effect of staining techniques and multiple firings on the translucency, color and biaxial flexural strength of advanced lithium disilicate ALD containing Virgilite crystals.

**Methods:**

Sixty-three discs of ALD (CEREC Tessera^®^) were divided into 3 groups based on staining techniques (*n* = 21); group CO (glaze only), group SC (single-step characterization), and group DC (double-step characterization). The discs were then subjected to either 2, 4, or 6 firing cycles, resulting in 9 groups (*n* = 7): COII, COIV, COVI, SCII, SCVI, DCII, DCIV, and DCVI. Relative translucency parameter (RTP), color change (ΔE), and biaxial flexural strength were measured, then discs were analyzed using SEM. Data were statistically analyzed using ANOVA, Bonferroni correction, and Spearman’s correlation (α = 0.05).

**Results:**

Repeated firing and staining techniques significantly affected translucency, color change, and biaxial flexural strength (*p* < 0.001). Translucency increased with firings, highest in CO and lowest in DC. ΔE increased with firings, highest for DC and lowest in CO. The biaxial flexural strength of the CO group remained stable across firing cycles, with no significant changes. The SC group, initially the weakest, showed a significant increase, reaching its peak after six cycles. The DC group had high strength in the fourth cycle, with a significant difference observed between the second and fourth cycles. By the sixth cycle, all groups showed comparable strength with no significant differences.

**Conclusions:**

Within the limitation of this study, firing cycles and staining techniques impact the properties of ALD. More firing cycles enhance translucency but increase color change. Repeated firing, particularly with the double-step characterization technique, significantly improved biaxial flexural strength up to the fourth cycle, demonstrating its superior performance over the single-step characterization technique.

## Background

All-ceramic restorations are highly valued for their excellent appearance and light transmission, simulating natural enamel and dentin. Various all-ceramic materials are available, with no single one being suitable for all clinical situations [1]. Among them, lithium disilicate is a top choice for highly esthetic restorations due to its superior biocompatibility, plaque resistance, and natural appearance as well as their strong mechanical properties [2, 3].

As chairside monolithic restorations gained prime interest due to the increasing value of time and the evolution of CAD/CAM technology making them more feasible [4], glass ceramic restorations have also advanced to meet the growing demand for high esthetics and longevity without lengthy and time-consuming laboratory steps. One of the latest versions of glass-matrix ceramics is the advanced lithium disilicate (ALD) glass ceramic, such as CEREC Tessera^®^, which contains Virgilite crystals (Li_0.5_Al_0.5_Si_2.5_O_6_) within a glassy zirconia matrix. ALD offers both strength and esthetics with a notably short firing time of just 4 min and 30 s in an induction chairside furnace [5–8].

Although the microstructure of dental ceramics is the primary factor in determining their translucency and overall esthetics, translucency is also influenced by several other factors, including the presence of pores, secondary phase components, additives, refractive index and light scattering from the surface of the material [9–12]. Moreover, achieving an esthetically pleasing restoration relies heavily on effective shade matching, which is influenced by the ceramic material type, thickness, use of colorants, the effects of repeated firings and firing cycles [13–15]. While esthetic qualities are crucial, the mechanical properties of a material also contribute to the overall performance of a restoration. That’s why the choice of ceramic material, microstructure, fabrication technique, thickness, firing temperature, surface treatments, heat treatments, and the presence of flaws like cracks and voids introduced during processing and handling of the ceramic restoration or any surface or subsurface damage resulting from rotary instrument during intraoral adjustments is key in determining the strength performance and durability of the restoration [16–26].

Despite the common suggestion by many manufacturers that a single firing of monolithic lithium disilicate will yield the desired optical and structural properties, in clinical practice, there are instances where shade and color modifications to the restoration are necessary before its final cementation. This is crucial to achieve a perfect match that satisfies both the doctor and the patient. Consequently, additional cycles of firing may be required in such cases and using color paste is often necessary for corrections. This characterization step can be done during the crystallization step for conventional lithium disilicates or during the matrix firing for ALD. Alternatively, it may also be completed as a separate step [27, 28]. However, these subsequent firings can potentially change the material’s physical, optical, and mechanical properties [12, 15, 27–38].

To address these challenges, this study aimed to evaluate the effect of repeated firings and different staining techniques on the translucency, color and biaxial flexural strength of Virgilite-based advanced lithium disilicate material. The null hypothesis posited that the translucency, color, and biaxial flexural strength of the Virgilite-based advanced lithium disilicate material would remain unaffected by repeated firing cycles and the application of different staining techniques.

## Materials and methods

### Materials

The materials used in this study, composition, manufacture and lot number are shown in (Table 1).

**Table 1:** Material used in this study with its composition, manufacturer and lot number

Description	Composition	Manufacturer	Lot number
Advanced Lithium disillicate glass ceramic (CEREC Tessera^®^)	40–50%Glass zirconia matrix, 40%lithium disilicate (Li₂Si₂O₅),5% Virgilite (LiAlSiO_6_),5% lithium phosphate (Li₃PO₄)	Dentsply Sirona, Hanau -Wolfgang, Germany	16,013,709
Spray glaze (Universal Spray Galze Fluo)	Silicate glass, isopropyl alcohol, isobutane propellant, fluorescing agent	Dentsply Sirona Hanau-Wolfgang, Germany	A0952
Stain paste (Universal Stain)	ceramic oxides and coloring metal oxides	Dentsply Sirona Hanau-Wolfgang, Germany	20,001,490

### Sample grouping

Power analysis was conducted by adopting an alpha level of 0.05, a beta of 0.2 (i.e., power = 80%), and an effect size (f) of 0.596, calculated based on the results of a previous study [27, 28]. The predicted sample size (n) was a total of 63 samples, with 21 samples per group and 7 samples per subgroup. Sample size calculation was performed using G*Power version 3.1.9.7.

Sixty-three Virgilite-containing lithium disilicate discs (12 mm diameter, 1.2 mm average thickness) were constructed from CEREC Tessera^®^ CAD blocks (C14 MT A2, Dentsply, Sirona). The discs were allocated into 3 main groups according to different staining (*n* = 21): Group CO: (control group): glaze only, as manufacturer avoiding firing without glaze in the clinical practice, group SC: (single step characterization): glaze + stain in 1 cycle and group DC: (double step characterization): glaze 1 cycle and stain 1 cycle. Each technique of characterization (CO, SC and DC) had their samples subjected to 2, 4, or 6 firing cycles, resulting in 9 groups: COII, COIV, COVI, SCII, SCIV, SCVI, DCII, DCIV, and DCVI.

**Fig. 1:**
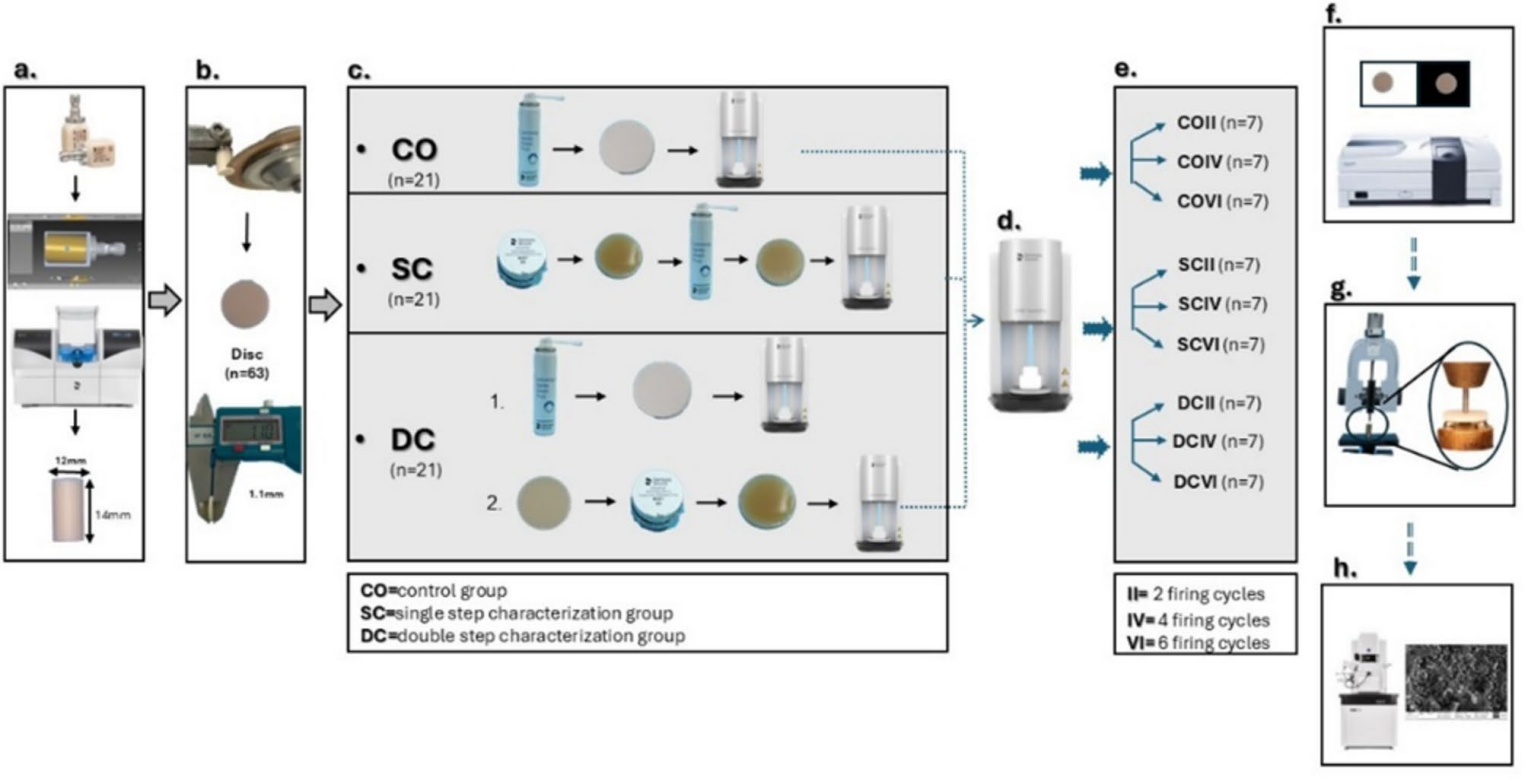
Schematic diagram for grouping and experimental design **a**. Cylinder preparation by designing then milling, **b**. Disc production using Isomet, **c**. Staining techniques, **d**. Firing using SpeedFire furnace, **e**. Repeated firing of each group, **f**. Color and translucency measurement using spectrophotometer, **g**. Biaxial flexural strength measurement using piston on 3 balls universal testing machine, **h**. Microstructural analysis by field emission scanning electron microscope (FE-SEM)

The smallest number of firings was 2, which is the minimal number necessary for characterization of the DCII group. The remaining firings (4 and 6) were designed to replicate future staining firings for hypothetical characterization adjustments [27, 28]. Sample preparation, sample grouping and distribution are summarized in (Fig. 1).

### Discs production

A cylinder of 12 mm in diameter was designed as an STL file with the designing software (inLab, Dentsply Sirona, Hanau -Wolfgang, Germany), then milled with the 5-axis milling machine (CEREC MCXL, Dentsply Sirona, Hanau -Wolfgang, Germany) under copious amount of coolant (Fig. 1a). Discs were sectioned from each cylinder using a linear precision saw (IsoMet 4000, BUEHLER LTD, USA), yielding 8 to 9 discs per cylinder with a thickness of 1.2 ± 0.2 mm [8, 27, 28, 39].

The discs were manually polished sequentially using three different grits of silicon carbide abrasive paper: 600 grit, 800 grit, and 1000 grit. Two uniform strokes were applied in the same direction on a single surface of each disc. This polishing protocol was adopted to ensure a smooth and consistent surface [8]. The dimensions of the discs were verified using a digital caliper and any defective discs were discarded (Fig. 1b). Discs were cleaned in an ultrasonic cleaner with ethanol for 1 min to eliminate any remaining residue on the surface and dried thoroughly [39]. Ceramic discs were randomly distributed into 3 groups (*n* = 21).

### Discs staining and glazing

Ceramic discs were subjected to one of the subsequent procedures according to grouping (Fig. 1c):

Control (CO group): only glazing was applied to create an even whitish layer after drying. The spray bottle was shaken for 20 s before application and its nozzle was held 12 mm away from the disc [39].

The single-step characterization (SC group): stain and glaze were applied simultaneously before firing. Stain paste was first adjusted to the desired consistency with porcelain liquid using a non-metallic plastic spatula. A thin, even layer was then applied to the polished disc surface with a brush in one direction, followed by a spray glaze over the stained surface before firing.

The double-step characterization (DC group): first, a universal spray glaze was applied to the polished discs and fired in the (CEREC SpeedFire furnace Dentsply Sirona, Hanau -Wolfgang, Germany) then cooled to room temperature. Next, stain paste was applied to the glazed discs and fired again [27, 28].

### Samples firing

Discs from each characterization group were further divided into three subgroups: 2 firing cycles (II), 4 firing cycles (IV), and 6 firing cycles (VI), with all undergoing the same firing program without adding any new stain layer (Fig. 1e). For the DC group, however, the first firing cycle was a glaze firing, the second was a stain firing, and subsequent firings were conducted without any added characterization layers.

First, each disc was placed on the firing pad which is placed on the honeycomb tray. Then, the whole assembly was inserted inside of the speed fire glaze program for CEREC Tessera^®^ was selected (Fig. 1d). The samples were fired 1 disc at a time following the manufacturer’s recommendation and bench cooled to room temperature.

### Measurements

#### Color and translucency measurement

The color and translucency measurements were conducted only on samples from groups COVI, SCVI, and DCVI (*n* = 21) for standardization [27].

Each sample had its color and translucency verified after different numbers of firing: 2, 4 and 6.

One measurement was made per sample using a double beam reflectance spectrophotometer (Cary 5000 UV-Vis-NIR Spectrophotometer; Agilent Technologies). Calibration of the spectrophotometer was checked prior to each measurement session as recommended by the manufacturer. Color measurements were performed under D65 illuminant (Fig. 1f) [27, 28].

#### Relative translucency parameter

The color difference between the samples on black background and white background is calculated and established as a translucency parameter Two backgrounds were used: a white tile (CIE L* = 98.35, a* = − 0.2, and b* =1.16) and a black tile (CIE L* = 2.88, a* = − 0.12, and b* = − 1.09) relative to the CIE standard illuminant D65.

Then relative translucency parameter (RTP) was obtained by the following equation:

$$\:RTP=\sqrt{{\left({l}_{w}^{*}-{l}_{b}^{*}\right)}^{2}+{\left({a}_{w}^{*}-{a}_{b}^{*}\:\right)}^{2}+{\left({b}_{w}^{*}-{b}_{b}^{*}\right)}^{2}}$$

Where the subscripts W and B refer to the color coordinates over the white and black background, respectively.

L* denotes the lightness, which ranges from zero (black) to 100 (white). a* and b* are the chromaticity coordinates in the red–green axis (− a* = green and + a* = red) and the yellow–blue axis (− b*= blue and + b* = yellow), respectively.

### Color change

The color difference between the compared colors is expressed in ∆E units. The total color difference, according to L*, a*, b* coordinates, is calculated as shown in the equation:

$$\:\varDelta\:{E}^{\text{*}}=\sqrt{{\left(\varDelta\:{l}^{\text{*}}\right)}^{2}+{\left(\varDelta\:{a}^{\text{*}}\right)}^{2}+{\left(\varDelta\:{b}^{\text{*}}\right)}^{2}}\:$$

#### Biaxial flexural strength measurements

All samples from each of the 9 groups (*n* = 7) were tested individually using a piston-on-three-ball universal testing machine (Fig. 1g). The apparatus included 3 steel balls 2.5 mm diameter arranged in a 12 mm diameter circle, 120° apart, and a piston with a 1.87 mm diameter round end. The samples were placed on the balls with the characterization side up facing load application simulating clinical conditions and pressed by the piston at a 0.5 mm/min crosshead speed until fracture. The force at failure (N) was recorded and used to calculate the biaxial flexural strength (σ, MPa) using the following equations:

1 $$\:\sigma\:=\frac{-0.2378P\left(X-Y\right)}{{b}^{2}}$$

2 $$\:X=\left(1+v\right)\text{ln}\left[{\left(\frac{r2}{r3}\right)}^{2}\right]+\left[\left(\frac{1-v}{2}\right){\left(\frac{r2}{r3}\right)}^{2}\right]$$

3 $$\:Y=\left(1+v\right)\left(1+\text{ln}\left[{\left(\frac{r1}{r3}\right)}^{2}\right]\right)+\left[\left(1-v\right){\left(\frac{r1}{r3}\right)}^{2}\right]$$

where P is a load at fracture (N), b is the sample thickness (mm), υ is Poisson’s ratio (0.2 for glass ceramics), r1 is the radius of the support circle (mm.), r2 is the radius of the loaded area (mm.), and r3 is the radius of the sample (mm).

### Microstructural analysis

One representative sample from each group was etched for 30 s by 4.5% hydrofluoric acid (HF) (Porcelain etchant; Bisco) [39], rinsed with 96% ethanol, dried, gold sputtered (Desk Sputter Coater; Vac Techniche Ltd) for 120 s to reduce scanning faults and image artifacts, and evaluated by using a field emission scanning electron microscope (FEI Quanta FEG250-FEI-USA) at different magnifications (×10000, ×30000, and ×50000) (Fig. 1h).

### Statistical analysis

Numerical data were presented as mean and standard deviation (SD) values. The normality assumption was validated by checking the data distribution and using Shapiro-Wilk’s test. Optical properties were analyzed using mixed model two-way ANOVA, while flexural strength data were analyzed using two-way ANOVA. Comparisons of simple effects were made using the pooled error term of the two-way model, and p-values were adjusted for multiple comparisons utilizing Bonferroni correction. The correlation was analyzed using Spearman’s rank-order correlation coefficient. The significance level was set at *p* < 0.05. Statistical analysis was performed with the R statistical analysis software version 4.3.1 for Windows.

## Results

### Relative translucency parameter

The study found that the translucency of advanced lithium disilicates (ALD) is significantly affected by both the number of firing cycles and the staining, with a significant interaction between them (*p* < 0.001).

The CO group had the highest translucency, followed by SC group, then DC group, all improving with more firing cycles (*p* < 0.001) (Table 2).

**Table 2:** Intergroup comparisons, mean and standard deviation (SD) values of relative translucency parameter (RTP) for different staining techniques and number of firing cycles

	Control group (CO)	Single step characterization (SC)	Double step characterization (DC)	*p*-value
Two firing cycles (II)	16.45 ± 0.18^A, c^	10.76 ± 0.16^B, c^	9.44 ± 0.15^C, c^	< 0.001*
Four firing cycles (IV)	18.37 ± 0.26^A, b^	11.85 ± 0.22^B, b^	10.49 ± 0.16^C, b^	< 0.001*
Six firing cycles (VI)	18.87 ± 0.19^A, a^	12.33 ± 0.20^B, a^	10.96 ± 0.20^C, a^	< 0.001*
p-value	< 0.001*	< 0.001*	< 0.001*	

Different uppercase letters indicate a statistically significant difference within the same horizontal row and lowercase letters indicate a statistically significant difference within the same vertical column row *; significant (*p <* 0.05), ns; non-significant (*p* > 0.05)

### Color change

The results of the color change (ΔE) assessment showed significant differences in color influenced by the staining techniques and the number of firing cycles independently (*p* < 0.001). While their interaction was statistically insignificant (*p* = 0.305).

Within each staining group, increasing the number of firing cycles led to a progressive increase in color change (*p* < 0.001).

Across different firing cycles, the DC technique consistently resulted in the highest color change, followed by SC and CO (*p* < 0.001) (Table 3).

**Table 3:** Intergroup comparisons, mean and standard deviation (SD) values of color change (ΔE) for different staining techniques within different firing cycles

	Control group (CO)	Single step characterization (SC)	Double step characterization (DC)	*p*-value
From 2 to 4 firing cycles(ΔE 2x-4x)	2.12 ± 0.14^C, b^	2.59 ± 0.13^B, b^	2.87 ± 0.12^A, b^	< 0.001*
From 2 to 6 firing cycles (ΔE 2x-6x)	2.44 ± 0.17^C, a^	2.98 ± 0.17^B, a^	3.36 ± 0.13^A, a^	< 0.001*
p-value	0.012*	0.002*	< 0.001*	

Different uppercase letters indicate a statistically significant difference within the same horizontal row and lowercase letters indicate a statistically significant difference within the same vertical column row *; significant (*p <* 0.05), ns; non-significant (*p* > 0.05)

### Biaxial flexural strength

The results of the biaxial flexural strength revealed a significant interaction between the technique of staining and repeated firing (*p* < 0.001). The biaxial flexural strength after the 2nd firing cycle was highest in DCII, followed by COII, then SCII. For the 4th firing cycle, DCIV showed statistically significant higher strength than COIV and SCIV. As for the 6th firing cycles, all different staining techniques showed similar strength (*p* = 0.284). Among different staining techniques, CO remained stable across different firing cycles(*p* = 0.806). SC, initially the weakest in SCII, improved in SCIV then finally SCVI matched subgroup DCVI. While the biaxial flexural strength in subgroup DCII started high, it peaked in DCIV, then it declined in DCVI to match others (Table 4).

**Table 4:** Intergroup comparisons, mean and standard deviation (SD) values of biaxial flexural strength (MPa) for different staining techniques within different firing cycles

	Control group (CO)	Single step characterization (SC)	Double step characterization (DC)	*p*-value
Two firing cycles (II)	206.75 ± 5.99Ba	169.03 ± 7.57Cb	224.81 ± 17.82Ab	< 0.001*
Four firing cycles(IV)	204.51 ± 4.28Ba	212.40 ± 9.12Ba	243.40 ± 5.81Aa	< 0.001*
Six firing cycles(VI)	208.95 ± 10.37Aa	218.40 ± 11.90Aa	215.78 ± 9.70Ab	**0.284ns**
**p-value**	**0.806ns**	< 0.001*	< 0.001*	

Different uppercase letters indicate a statistically significant difference within the same horizontal row and lowercase letters indicate a statistically significant difference within the same vertical column row *; significant (*p <* 0.05), ns; non-significant (*p* > 0.05)

### Scanning electron microscope (SEM) results

**Fig. 2:**
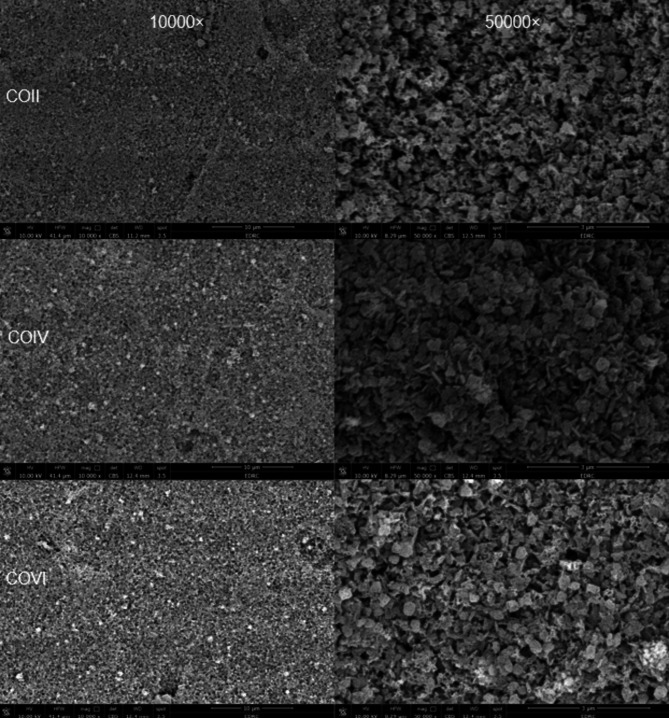
Representative scanning electron microscope images for Control groups at 10,000X (left-hand side) and 50,000x (right-hand side): COII: control group after 2 firing cycles; COIV: control group after 4 firing cycles; and COVI: control group after 6 firing cycles

**Fig. 3:**
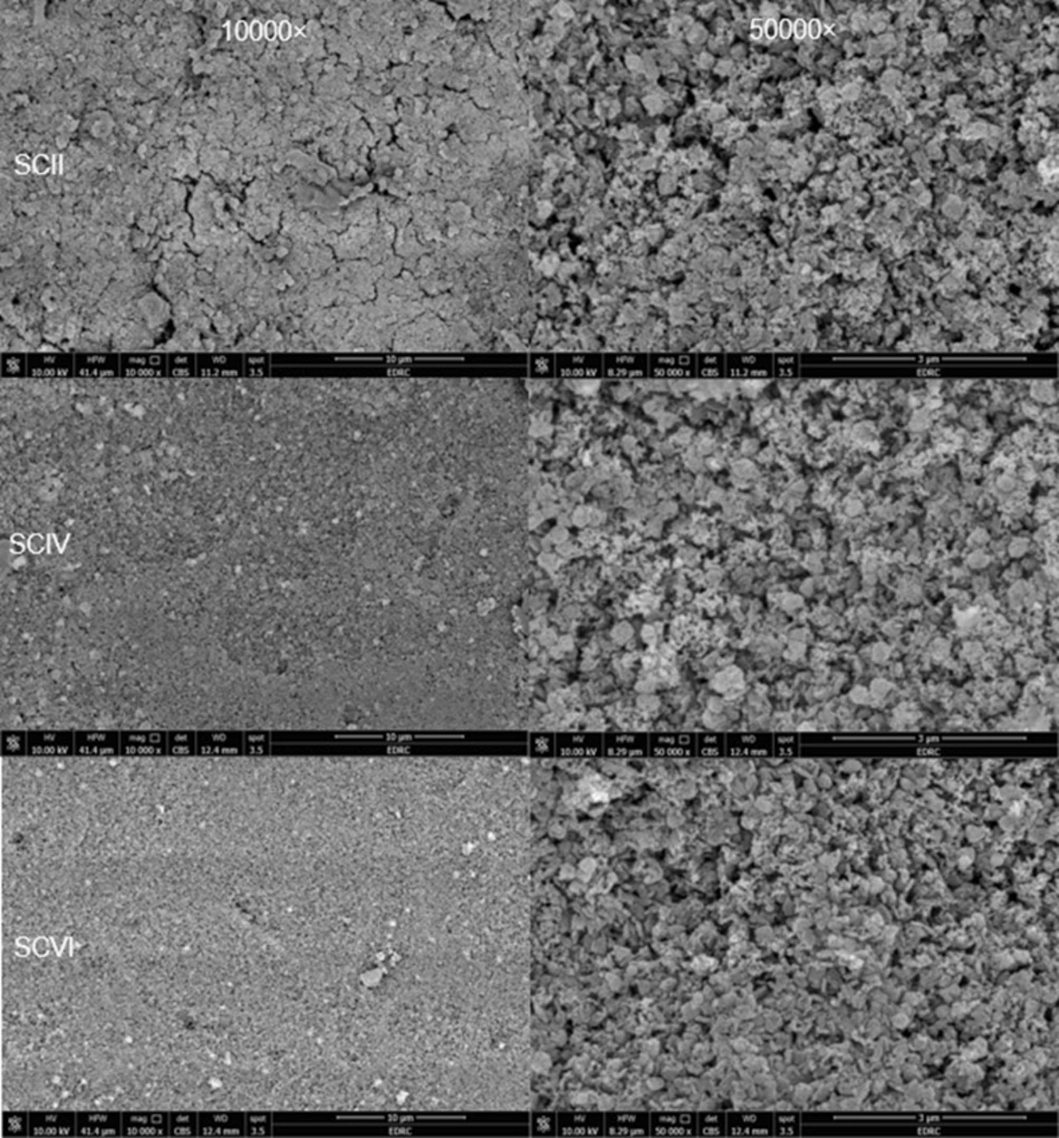
Representative scanning electron microscope images for single step characterization groups at 10,000X (left-hand side) and 50,000x (right-hand side): SCII: single step characterization group after 2 firing cycles; SCIV: single step characterization group after 4 firing cycles; and SCVI: single step characterization group after 6 firing cycles

**Fig. 4:**
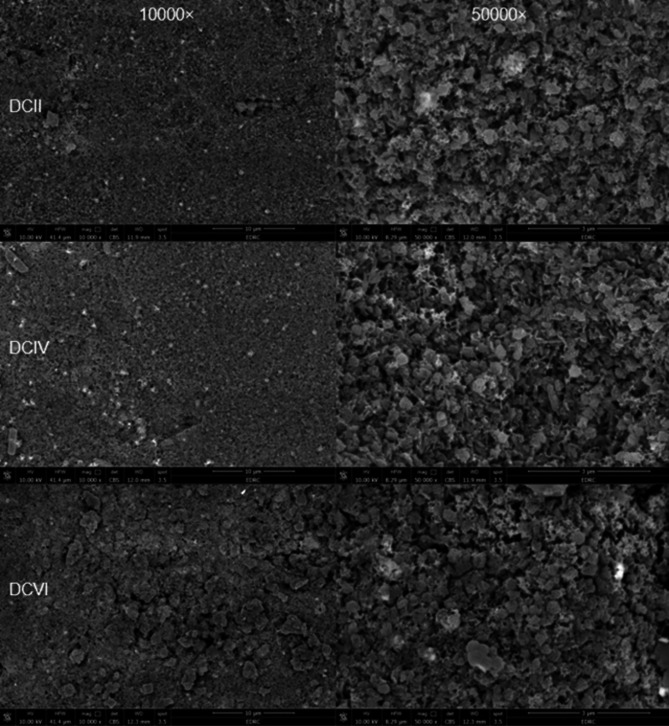
Representative scanning electron microscope images for double step characterization groups at 10,000X (left-hand side) and 50,000x (right-hand side): DCII: double step characterization group after 2 firing cycles; DCIV: double step characterization group after 4 firing cycles; and DCVI: double step characterization group after 6 firing cycles

The microstructure of etched advanced lithium disilicates is shown in (Figs. 2, 3 and 4).

The (Fig. 2) represents different firing cycles of the CO group where there was no change observed in the crystalline arrangement of the ALD.

In (Fig. 3), SCII showed cracks observed all over the disc. Meanwhile, SCIV (Fig. 3) showed crack healing along with an increase in crystalline structure of ALD specifically Virgilite crystals. More densification and crystal rearrangement were observed in (Fig. 3) representing SCVI. Regarding DCII (Fig. 4), the microstructure of ALD shows well-organized crystals with evidence of Virgilite crystals. In DCIV (Fig. 4) shows more densification in microstructure and an increase in the number of Virgilite crystals. On the other hand, (Fig. 4), representing DCVI, shows an increase in crystal size and shape becoming coarser.

## Discussion

Within the limitations of this study, the null hypothesis was totally rejected, as the results revealed that translucency, color change and biaxial flexural strength of Virgilite lithium disilicate are significantly affected by the number of firing cycles and the technique of staining (*p* < 0.001).

Regarding translucency, within each staining group, increasing the number of firing cycles led to a rise in translucency. The CO group consistently exhibited the highest translucency across all cycles, while the DC group showed the lowest. In this study, the RTP values ranged from 9.44 ± 0.15 for DCII to 18.87 ± 0.19 for COVI. According to international standards, the perceptibility threshold (PT) is set at ΔTP = 1.3, and the acceptability threshold (AT) at ΔTP = 4.4. This indicates that all values were perceptible and clinically unacceptable [40]. Translucency in dental materials is influenced by factors such as the crystalline-to-glass phase ratio, refractive index differences, crystal morphology, grain boundaries, porosity, secondary phases, additives, and surface light scattering [10]. Results from the current study suggest that firing the glaze and stain simultaneously form a single, integrated layer [28], facilitating light transmission and thus enhancing translucency [9, 10]. Conversely, when glaze is fired first, followed by stain in a separate cycle, distinct layers are created [28], introducing boundaries and refractive index differences that scatter light and reduce translucency in the DC group [10].

Additionally, the translucency parameter increases with the number of firing cycles, which may be attributed to a reduction in porosity volume with repeated firing [11, 12]. This enhancement may also be linked to the transformation of Virgilite crystals into quartz or quartz like phase after mandatory firing [39, 41], so it can be suggested that heat from each cycle promotes this transformation. Moreover, auto glazing during refiring also contributes, as reflow of the glass component fills surface defects, resulting in a smoother, more translucent and esthetically appealing surface in advanced lithium disilicates [16].

In agreement with our study, the study conducted by Zaghloul et al. [12] concluded that multiple firing cycles increase the translucency parameter of lithium disilicate ceramic. As well as the study of Miranda et al. [27] investigating lithium disilicates, IPS Emax CAD, supported the CO group results having the highest translucency in all groups regardless of the number of firing cycle also showing the COVI having the highest TP of all groups.

On the other hand, Rizk et al. [30] results were in disagreement with the present study concluding that the translucency of ALD decreases with repeated firing. The disparities in findings are likely attributable to the use of different furnace Programat EP 5000 hence different cycle duration and heat distribution, the absence of glaze layer recommended by the manufacturer and the use of TP00 instead of TP for assessment of translucency.

The results of the color change (ΔE) showed that within each staining group, increasing the number of firing cycles led to a progressive increase in color change. Across different firing cycles, the DC consistently resulted in the highest color change, while the CO the lowest. In this study, the ΔE values ranged from 2.12 ± 0.14 for CO (ΔE II-IV) to 3.36 ± 0.13 for DC (ΔE II-VI). According to international standards, the perceptibility threshold (PT) is set at ΔE = 1.2, and the acceptability threshold (AT) at ΔE = 2.7 [40, 42]. This indicates that the CO value may be perceptible but still acceptable, whereas the higher ΔE in DC slightly exceeds the AT, suggesting potentially noticeable and less clinically acceptable color variation.

The CO group, without stain application, showed the lowest ΔE values, likely due to the absence of extrinsic colorants that typically contribute to color changes after firing [14, 15]. The DC group, on the other hand, exhibited greater color change compared to the SC group. This can be attributed to the separate firing of stain and glaze in the DC group, which created a multilayered structure [28] that may significantly influence the final color of the restoration [43].

The finding that color change increases with repeated firing is consistent with the previously conducted studies on different lithium disilicates [14, 15, 30] attributing the increase in ΔE with repeated firing to the color instability of metal oxides during firings, changes in surface colorants, pigment breakdown, and the resulting alteration of light refraction.

Concerning the biaxial flexural strength, the results revealed that strength was significantly affected by staining techniques up to the 4th firing cycle while the 6th firing cycle showed no significant change in all groups, whereas the number of firing cycles impacted strength within each characterization technique except for the control group there was no statistically significant changes in strength with repeated firing cycles.

Regarding the staining groups, the results revealed that SCII had the lowest strength of all groups. It may be suggested that the simultaneous application of glaze and stain may affect heat flow to the disc during matrix firing, which could hinder the heat’s ability to reduce fissures from the milling process [20, 44]. Additionally, firing stains with glaze may trap porosities, leading to voids that act as stress concentrators and increase the risk of fractures at lower stress levels [21]. These suggestions were supported by the presence of cracks and fissures by the SEM analysis in (Fig. 3a).

On the other hand, the results of the DC group showed an initial higher strength over other characterization group. This can be interpreted as a consequence of the accumulation of desired residual compressive stresses from applying two separate layers on the disc surface. This process enhances the compressive stresses more effectively than the single layer. As a result, the force required to initiate a critical crack increased improving the material’s flexural strength and fracture toughness [18, 26]. Moreover, separate firing allows each of the glaze and stain layers to cool slowly, dissipating unfavorable tensile residual stresses resulting from the mismatch in thermal expansion between ceramic, glaze, and stain [22, 25, 26]. This may explain the cracks observed in SCII and their absence in COII or DCII (Fig. 3a). Also, the presence of a distinct glaze layer fired before stain application allowed the glaze layer to flow and seal defects improving the disc strength [16].

Regarding the effect of repeated firing on each characterization group, the result showed that the strength of the CO group was stable across different firing cycles which was supported by multiple studies [32–35, 37].

As for the SC group, biaxial flexural strength rapidly increased with repeated firing. This may be attributed to a healing effect in refired glazed advanced lithium disilicates, where the glaze flows, sealing surface defects and enhancing the material’s kinetic energy. As the glass component reflows, it fills flaws, smooths sharp edges, reduces defect depth, and initiates a crack-healing process that dominates the ceramic’s fracture behavior [16, 24]. This repeated firing may act similarly to an annealing treatment [17], as extended exposure to high temperatures [30] leads to defect closure [24], possibly explaining the crack disappearance observed in (Fig. 3b and c).

The results of the DC group across different firing cycles witnessed an increase in strength followed by a decrease. This reduction can be attributed to repeated firing, which increased crystalline content and formed new phases with irregular particle sizes, inducing localized stress and disrupting matrix-crystal interactions. This resulted in coarse, uneven crystal patterns prone to cracking. Mismatched thermal expansion coefficients between phases further weakened material cohesion [25, 30, 36]. SEM analysis revealed increased crystal coarseness with more firing cycles (Fig. 4a, b and c).

Moreover, the finding that all characterization groups reached similar, relatively high strength at six firing cycles may be due to the role of temperature in ceramic firing. Repeated high-temperature exposure reduces glass viscosity, increasing molecular mobility and minimizing structural weaknesses from initial firings, resulting in uniform strength across groups [19].

Although the biaxial flexural strength of all tested samples was below the 700 MPa claimed by the manufacturer [6], it exceeded the minimum threshold of 100 MPa required for monolithic single-unit anterior and posterior restorations [36].

These findings were in disagreement with the results of the study by **Miranda et al.** [28] concerning the staining techniques. The difference in results can be attributed to the different types of ceramic used, along with the use of a different type of stain creating an amorphous glass layer lowering the biaxial flexural strength.

Due to the novelty of the material, there was not enough literature on the combination of different staining techniques and repeated firing cycles.

### Clinical implications

Based on our findings, the single-step characterization technique should be prioritized in cases where aesthetics is of utmost importance. This method demonstrates the best optical properties, making it ideal for applications where appearance is a key consideration. On the other hand, the double-step characterization technique is more suitable for situations where enhanced mechanical performance is essential, as it offers superior initial strength. Regarding the number of firing cycles, it is recommended to limit the cycles to balance both aesthetic quality and clinically acceptable mechanical strength. This approach ensures that the final product maintains an optimal balance between appearance and durability, which is critical for clinical success.

Limitations of this study include using flat discs instead of tooth-shaped restorations, which may limit clinical relevance. Aging was not considered, though thermal changes in the oral cavity could influence results. Additionally, surface roughness, which affects restoration outcomes, was not assessed. This study focused solely on advanced lithium disilicate materials. Future studies should include comparative analyses of firing effects on lithium disilicate versus other ceramics to broaden the applicability of findings, as well as use anatomically accurate samples. The translucency and final color of ceramic restorations are influenced by the type of cement used in clinical practice. Future studies could evaluate how different types of resin cements impact the optical properties of these materials after multiple firing cycles, which may provide valuable insights into the long-term performance of ceramic restorations in clinical settings.

Furthermore, incorporating thermocycling and fatigue testing in future research would help provide a deeper understanding of the long-term mechanical performance and durability of these ceramics. To further investigate the impact of glazing and staining on the optical and mechanical properties, future studies should include measurements of surface roughness before and after repeated firings. This data would clarify how surface roughness influences translucency and color change, helping to explain the observed differences in these properties.

## Conclusion

Within the limitations of this in vitro study, the following conclusions could be drawn:

1. Firing cycles and staining techniques impact the properties of lithium disilicate containing Virgilite crystal.
2. More firing cycles enhance translucency and strength up to a point but increase color change.
3. Single step characterization offers the best optical properties, while double-step characterization provides the best initial strength.
4. Limiting the number of firing cycles can help maintain the mechanical integrity of lithium disilicate restorations while still achieving the desired aesthetic results.

## Data Availability

The datasets used and analyzed during the current study are available from the corresponding author on reasonable request and will be sent.

## Abbreviations

ALD: Advanced lithium disilicate
CO: Control
SC: Single step characterization
DC: Double step characterization
RTP: Relative translucency parameter
PT: Perceptibility threshold
AT: Acceptability threshold
